# SIRT1 Protects Against Apoptosis by Promoting Autophagy in the Oxygen Glucose Deprivation/Reperfusion-Induced Injury

**DOI:** 10.3389/fneur.2019.01289

**Published:** 2019-12-05

**Authors:** Qiannan Ren, Zhiying Hu, Yuting Jiang, Xiaoning Tan, Benson O. A. Botchway, Nashwa Amin, Gaoping Lin, Yu Geng, Marong Fang

**Affiliations:** ^1^Institute of Neuroscience, Zhejiang University School of Medicine, Hangzhou, China; ^2^Department of Obstetrics and Gynecology, Hangzhou Red Cross Hospital, Hangzhou, China; ^3^Department of Neurology, Zhejiang Provincial People's Hospital, Hangzhou, China; ^4^People's Hospital of Hangzhou Medical College, Hangzhou, China

**Keywords:** silent information regulator 1(SIRT1), apoptosis, autophagy, oxygen and glucose deprivation/reperfusion (OGD/R), cerebral ischemia/reperfusion (I/R) injury

## Abstract

Silent information regulator 1 (SIRT1) contributes to cellular regulation. Previous studies have reported SIRT1 to be abnormally expressed in the ischemic penumbra of cerebral ischemia/reperfusion (I/R) injury rat model. We investigated the effect of SIRT1 on oxygen and glucose deprivation/reperfusion (OGD/R) cell injury. Over-expressed or silenced SIRT1 pheochromocytoma 12 (PC12) cells were exposed to an *in-vitro* OGD/R injury. Western blot, TUNEL staining and immunofluorescence analyses were performed to assess apoptosis and autophagy. We found autophagy and apoptosis to be up-regulated and down-regulated, respectively, following the over-expression of SIRT1 in the OGD/R-induced PC12 cells. We also found the silencing of SIRT1 to culminate in the down-regulation and up-regulation of autophagy and apoptosis, respectively. On the basis of our results, we surmise that SIRT1 can promote autophagy and inhibit apoptosis *in-vitro*, and thus exhibit potential neuroprotection against OGD/R-induced injury. This could facilitate in the development of therapeutic approaches for cerebral I/R injury.

## Introduction

Stroke is a common type of cerebrovascular disease and results in about 6,000,000 deaths annually (1). Patients with acute stroke from vessel occlusion could be treated with intravenous thrombolysis (IVT); however, the treatment efficacy is highly time-dependent (2). The ineffectualness of the current treatments for stroke necessitates new effective therapeutic methods. In ischemic stroke, neurons in the brain are damaged as a result of the occlusion of oxygen and energy. Following the restoration of blood, a series of reactions instigate ischemia/reperfusion (I/R) injury, eventually culminating in neuronal death (3). Hence, the amelioration of ischemic stroke could lie in curtailing the death rate of nerve cells while protecting the integrity of nerve function following cerebral I/R injury.

Oxygen and glucose deprivation-reperfusion (OGD/R) cell model is commonly used to simulate the state of neuronal I/R injury that results in neuronal apoptosis (4). Apoptosis, a process of programmed cell death, has been reported to be involved in the loss of nerve cells in both stroke and neurodegenerative diseases (5). The B-cell lymphoma-2 (Bcl-2) family proteins are key regulatory molecules of apoptosis. The Bcl-2 family proteins are divided into two groups, anti-apoptotic(such as Bcl-2, Bcl-xl, Bcl-w, and Mcl-1) and pro-apoptotic(such as Bax, Bak, Bad, Bid, and Bim) (6). The fate of a cell depends on the interactions between these proteins and the outer mitochondrial membrane. Studies have reported these proteins to have a significant role in I/R injury (7).

The mammalian silent information regulator 2 homolog, SIRT1, is a member of the deacetylase protein family Sirtuin. SIRT1 is involved in the regulation of a variety of cellular stress responses such as inflammation, autophagy and apoptosis (8). In the central nervous system diseases including cerebral ischemia, traumatic brain injury, Alzheimer's disease and Parkinson's disease, SIRT1 has shown protective effects due to its functions in metabolism, stress resistance, and genomic stability (9, 10). The protective effect of SIRT1 is potentially mediated through proteins such as PI3K-AKT, AMPK, MAPK, and FOXO (11). Abnormal expression profile of SIRT1 and various autophagy-related proteins were found in the ischemic penumbra in a rat model of cerebral I/R (12).

Macroautophagy (hereafter “autophagy”) is a pathway of cellular waste degradation (13). Autophagy refers to a bilayer membrane structures called autophagosomes which contains the damaged organelles and fuses with lysosomes to generate autolysosomes in which cellular constituents are broken down by acid hydrolases (14). Autophagy is involved in cell growth, survival, and differentiation and such can have disparate effects in anti-cancer, anti-aging, anti-microbial defense, and neuroprotection (15). Following the first investigation of autophagy in yeast, more than 30 genes related to autophagy have since been discovered and named autophagy related genes (*Atg*) (16). It has been demonstrated that modulating autophagy could confers protection against I/R injury both *in vivo* and *in vitro* (17, 18).

To explore the role of SIRT1 in I/R injury, a combination of SIRT1 over-expression or knock-down and OGD/R treatment were employed in PC12 cells. Several experimental analyses were then conducted to determine the extent of autophagy and apoptosis while also ascertaining the role of SIRT1 in OGD/R treated PC12 cells.

## Materials and Methods

### Experimental Design

Five experimental groups were assigned: Normal group (cells not transfected with any plasmid), SIRT1 Control group (cells transfected with SIRT1 control plasmid), SIRT1 Over-expression group (cells transfected with SIRT1 over-expressed plasmid), SIRT1 shRNA group (cells transfected with plasmid encoding SIRT1-targeted shRNA) and SIRT1 shRNA Control group (cells transfected with plasmid encoding SIRT1-targeted shRNA's scramble control). In each group, the corresponding plasmid was transfected to the cells 24 h prior to OGD/R.

### Cell Culture

PC12 cells (ATCC Mouse Cell) were thawed in 37°C water-bath and cultured in RPMI 1640 medium (Gibco) containing 10% fetal bovine serum (FBS) (Vistech) and penicillin-streptomycin mixture (1:1,000 dilution, Gibco) at 37°C/5% CO_2_. Pre-configured medium was stored in a 4°C refrigerator and pre-heated in a 37°C water-bath before use. The medium was changed daily; Cells were washed twice in 0.01 M phosphate buffer saline (PBS) (HyClone) and passaged by using 0.25% trypsin (Gibco) when the cell density was more than 80%.

### Establishment of Cell OGD/R Model

Cells at a density of 1 × 10^4^ cells per well were cultured in a pre-heated RPMI-1640 glucose-free medium (Gibco). The plate was put into an anaerobic culture bag (MGC) which included an anaerobic gas producing bag (MGC) and an anaerobic indicator (MGC). With no change in the indicator, the anaerobic culture bags containing 6-well plates were put into CO_2_ incubator for 0, 2, and 4 h. After deprivation of glucose and hypoxia, the anaerobic devices were removed, and the cells were cultured in a full culture medium at 37°C/5% CO_2_ for 24 h.

### Cell Viability Assay

Following OGD/R, cell viability was measured using the cell counting kit 8 (CCK8) (Bestbio). Briefly, cells were incubated in a medium containing 10% CCK-8 solution for 4 h at 37°C. The absorbance at 450 nm was determined using a microplate reader. On the basis of cell viability, the most suitable OGD time was chosen for subsequent experiments.

### SIRT1 Over-expression and Knockdown

The cells were seeded in a 6-well plate in advance. Plasmids were transfected based on the protocol of jetPRIME® *in-vitro* DNA & siRNA transfection reagent (Polyplus). Jetprime buffer (200 μl), plasmid (2 μg), and jetprime (4 μl) were mixed together. The transfection mixture was incubated at a room temperature for 10 min. Then, cells in the 6-well plate were incubated with transfection mixture (200 μl per well) for 4 h, after which the media was replaced with normal media. Puromycin solution (0.4 μl) (Solarbio, 10 mg/mL) was added to 2 mL medium to prepare the puromycin dilution. After the plasmid transfection, the cells in the SIRT1 shRNA group and SIRT1 shRNA Control group were selected with puromycin dilution for 24 h at CO_2_ incubator.

### TUNEL Staining

TUNEL staining was conducted using *in situ* cell death detection kit (Roche, 11684795910). Cells seeded on the coverslips were fixed in a 4% paraformaldehyde in 0.01 M PBS (pH 7.4) for 1 h at room temperature and permeabilized with 0.1% Triton X-100 in a 0.1% sodium citrate for 2 min on ice. Cells were washed with PBS thrice. After, the TUNEL reaction solution (50 μl) was added and the cells were incubated in a dark and humidified atmosphere for 1 h at 37°C. A mounting medium containing DAPI (VECTASHIELD, USA) was then added to the slides and covered with coverslips for observation. Slides were observed under a fluorescence microscope (Olympus BX61, Japan) at excitation/emission wavelengths of 547/570 nm (TRITC, red) and 360/460 nm (DAPI, blue). Images were taken at 200× magnification.

### Immunofluorescence Staining

Cells on the coverslips were fixed in a 4% paraformaldehyde for 20 min at room temperature in advance. The fixed cells were permeabilized with 0.2% Triton X-100 in 0.01 M PBS for 15 min. After blocking with 5% BSA in 0.01 M PBST, the fixed cells were incubated with rabbit anti-LC3B primary antibody (1:250 dilution, CST) in PBST at 4°C overnight. The cells were incubated with anti-rabbit Alexa Fluor594 secondary antibody (1:500, EARTHOX, USA) in 0.01 M PBST at room temperature for 1.5 h after washing every 5 min for three times with PBST. After incubating with secondary antibody, a mounting medium containing DAPI (VECTASHIELD, USA) was added to the slides and then covered with coverslips for observation. Slides were observed under a laser scanning confocal microscopy (Olympus FV1000, Japan) at excitation/emission wavelengths of 547/570 nm (TRITC, red) and 360/460 nm (DAPI, blue). Images were taken at 400× magnification. For negative controls, cells on the coverslips were incubated with only secondary antibody.

### Western Blot Analysis

Cell lysates were prepared by incubation in RIPA lysis buffer (Auragene) containing protease inhibitors (Roche) and phosphatase inhibitor (Roche). The protein concentrations were measured using BCA protein quantitative detection kit (Auragene). The sample protein was boiled at 100°C for 10 min in advance. Total protein (approximately 20 μg) was separated by electrophoresis (200 V) and transferred to polyvinylidene difluoride membrane (Immobilon) at 300 mA. The membrane was blocked with 5% skim milk for 3 h and incubated with primary antibodies in a 4°C refrigerator overnight. The following primary antibodies were used: rabbit anti-Atg5 antibody(1:1,000 dilution, Abcam), rabbit anti-LC3B antibody (1:1,000 dilution, CST), rabbit anti-GAPDH antibody (1:1,000 dilution, CST), rabbit anti-Bcl-2 antibody (1:400 dilution, Boster), rabbit anti-SIRT1 antibody (1:1,000 dilution, CST), rabbit anti-Bax antibody (1:400 dilution, Boster), rabbit anti-Beclin1 antibody (1:1,000 dilution, CST), and mouse anti-P62 antibody (1:1,000 dilution, Abnova). On the next day, the membrane was incubated with secondary antibodies at room temperature for 2.5 h after washing three times with TBST. The secondary antibodies were HRP-conjugated goat anti-rabbit antibody (1:5,000 dilution, Bioker) and HRP-conjugated goat anti-mouse antibody (1:5,000 dilution, Earth). The protein band was visualized using the ChemiDoc Touch Imaging System after incubating with enhanced chemiluminescence and quantified by Image Lab software. All experiments were triplicated.

### Statistical Analysis

Gray values of western blot results were analyzed with Image Lab software. TUNEL staining results were analyzed with Image J software. Immunofluorescence results were analyzed with Pro Image Plus. Statistical significance (*P* < 0.05 was considered statistically significant, ^*^P < 0.05, ^**^P < 0.01, ^***^P < 0.001) was determined by using least significant difference (LSD) *post-hoc*-test in one-way analysis of variance (ANOVA) in SPSS 22.0 software and histograms were generated using GraphPad Prism 7. All data are presented as mean ± SEM (*n* = 3).

## Results

### OGD/R Treatment Induces PC12 Cells Death

**Figure 1:**
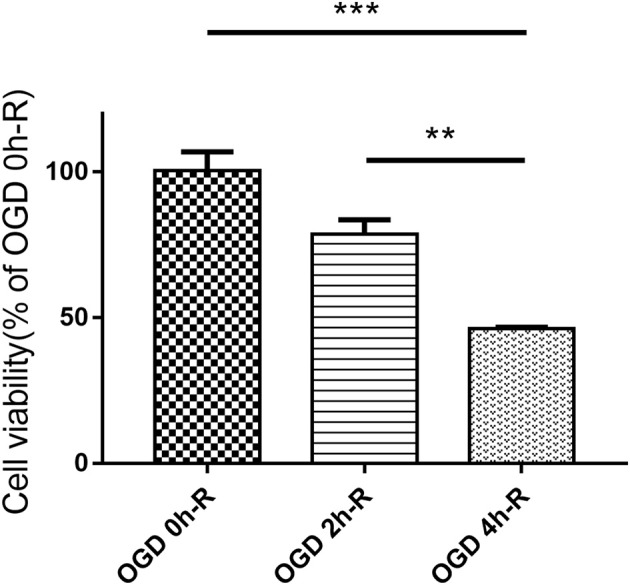
PC12 cells viability evaluated with CCK8 assay after treating with three different OGD times (0, 2, and 4 h). Values are expressed as mean ± SEM (*n* = 3). OGD 4 h/R vs. OGD 0 h/R, ***P < 0.001; OGD 4 h/R vs. OGD 2 h/R, **P < 0.01.

The effect of the three different OGD times (0, 2, and 4 h) followed by 24 h reperfusion on PC12 cell injury was evaluated using CCK8 assay. Cell viability did significantly decrease in the OGD 4 h/R-induced cells when compared to both the control group (OGD 0 h/R) (*n* = 3, *P* < 0.001) and the OGD 2 h/R-induced cells (*n* = 3, *P* < 0.01; Figure 1).

### SIRT1 Increased Bcl-2/Bax Ratio in OGD/R- Induced PC12 Cells

**Figure 2:**
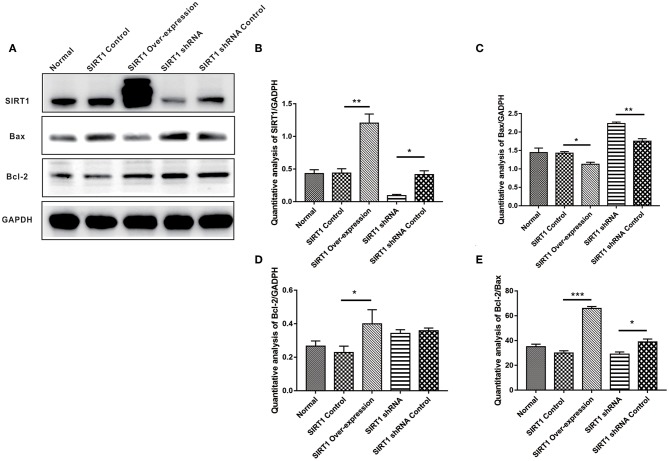
Effect of SIRT1 on OGD/R-induced cells apoptotic level. **(A)** Western blot results of SIRT1, Bax, Bcl-2, and GADPH. **(B)** Quantification analysis of SIRT1. Values are expressed as mean ± SEM (*n* = 3). SIRT1 Control vs. SIRT1 Over-expression, **P < 0.01; SIRT1 shRNA vs. SIRT1 shRNA Control, *P < 0.05. **(C)** Quantification analysis of Bax. SIRT1 Control vs. SIRT1 Over-expression, *P < 0.05; SIRT1 shRNA vs. SIRT1 shRNA Control, **P < 0.01. **(D)** Quantification analysis of Bcl-2. SIRT1 Control vs. SIRT1 Over-expression, *P < 0.05. **(E)** Quantification analysis of Bcl-2/Bax. SIRT1 Control vs. SIRT1 Over-expression, ***P < 0.001; SIRT1 shRNA vs. SIRT1 shRNA Control, *P < 0.05.

In assessing apoptosis, we found the Bcl-2 band for the SIRT1 Over-expression group to be strongly expressed when compared to the SIRT1 Control group (*n* = 3, *P* < 0.05), while the Bax band showed a weak expression (*n* = 3, *P* < 0.05). There was a strong Bax band expression in the SIRT1 shRNA group in comparison to the SIRT1 shRNA Control group (*n* = 3, *P* < 0.01); there was no significant change in the Bcl-2 expression (*n* = 3, *P* > 0.05; Figures 2C,D). The Bcl-2/Bax ratio was significantly increased in the SIRT1 Over-expression group than the SIRT1 Control group (*n* = 3, *P* < 0.001; Figure 2E). Also, the Bcl-2/Bax ratio was significantly decreased in the SIRT1 shRNA group than the SIRT1 shRNA Control group (*n* = 3, *P* < 0.05; Figure 2E).

### SIRT1 Decreases the Number of Apoptotic Cells in OGD/R-Induced PC12 Cells

**Figure 3:**
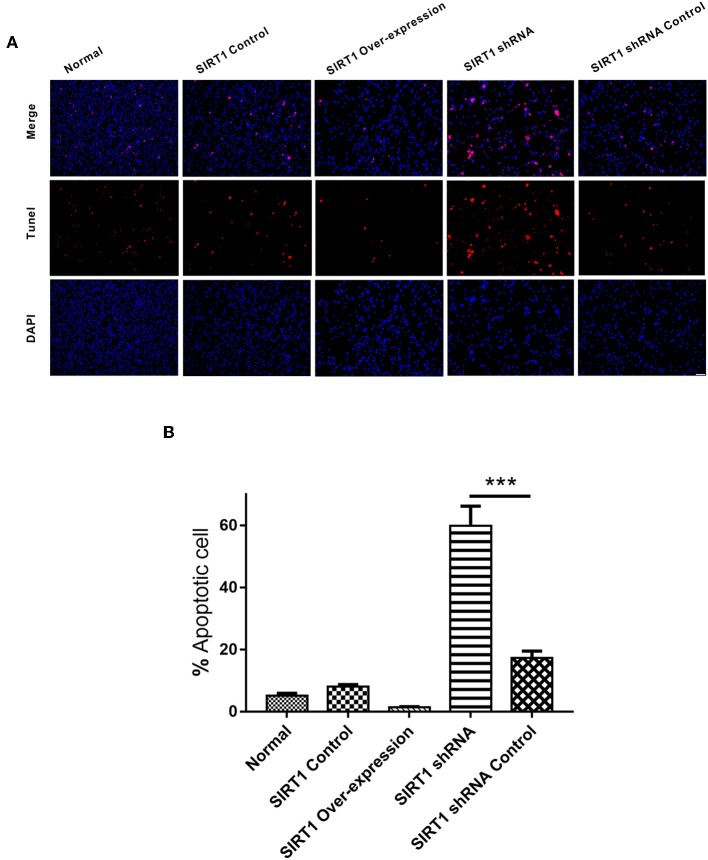
Apoptosis detected by TUNEL staining in each group. **(A)** Representative images of TUNEL-positive cells (red) and DAPI (blue). Both images are merged at the top panel. Scale bar = 100 μm. **(B)** Quantification analysis of TUNEL-positive cells. Values are expressed as mean ± SEM (*n* = 3). SIRT1 shRNA vs. SIRT1 shRNA Control, ***P < 0.001.

In the TUNEL staining analysis, there was no significant difference on the numbers of apoptotic cells between the SIRT1 Over-expression group and the SIRT1 Control group (*n* = 3, *P* > 0.05). The proportion of apoptotic cells in the SIRT1 shRNA group was significantly higher when compared to the SIRT1 shRNA Control group (*n* = 3, *P* < 0.001; Figure 3B).

### SIRT1 Alters ATG5, P62, Beclin-1 Expressions and LC3-II/I Ratio in OGD/R- Induced PC12 Cells

**Figure 4:**
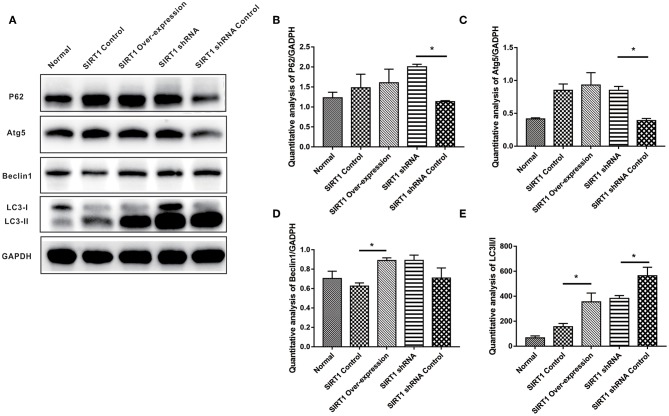
Effect of SIRT1 on OGD/R-induced cells autophagic level. **(A)** Western blot results of P62, Atg5, Beclin1, LC3, and GADPH. **(B)** Quantification analysis of P62. Values are expressed as mean ± SEM (*n* = 3). SIRT1 shRNA vs. SIRT1 shRNA Control, *P < 0.05. **(C)** Quantification analysis of Atg5. SIRT1 shRNA vs. SIRT1 shRNA Control, *P < 0.05. **(D)** Quantification analysis of Beclin1. SIRT1 Control vs. SIRT1 Over-expression, *P < 0.05. **(E)** Quantification analysis of LC3-II/I. SIRT1 Control vs. SIRT1 Over-expression, *P < 0.05; SIRT1 shRNA vs. SIRT1 shRNA Control, *P < 0.05.

We examined the expression levels of autophagy indices (P62, Atg5, Beclin-1, and LC3-II/I), and the representative images of Western blot of these proteins can be seen in Figure 4A. Both P62 and Atg5 expressions showed stronger bands in the SIRT1 shRNA group than the SIRT1 shRNA Control group (*n* = 3, *P* < 0.05). No significant difference in both P62 and Atg5 expressions was observed between the SIRT1 Over-expression group and the SIRT1 Control group (*n* = 3, *P* > 0.05; Figures 4B,C). Beclin-1 expression for the SIRT1 Over-expression group showed a stronger band when compared to the SIRT1 Control group (*n* = 3, *P* < 0.05), while there was no significant difference in Beclin-1 expression between the SIRT1 shRNA group and the SIRT1 shRNA Control group (*n* = 3, *P* > 0.05; Figure 4D). LC3-II/I was significantly higher in the SIRT1 Over-expression group than the SIRT1 Control group (*n* = 3, *P* < 0.05), while the LC3-II/I in the SIRT1 shRNA group was significantly lower than the SIRT1 shRNA Control group (*n* = 3, *P* < 0.05; Figure 4E).

### SIRT1 Increased the Formation of LC3 Dots in OGD/R- Induced PC12 Cells

**Figure 5:**
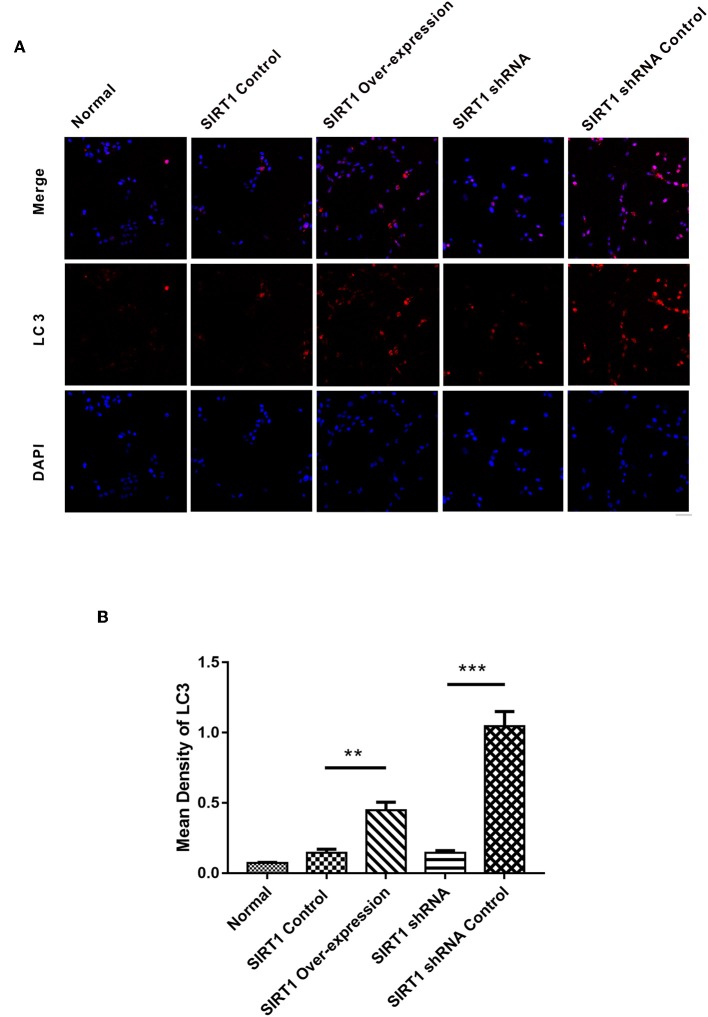
Fluorescence microscopic images of LC3 in each group. **(A)** Representative images of LC3 (red) and DAPI (blue). Both images are merged at the top panel. Scale bar = 40 μm. **(B)** Quantification of LC3 punctae. Values are expressed as mean ± SEM (*n* = 3). SIRT1 Control vs. SIRT1 Over-expression, **P < 0.01; SIRT1 shRNA vs. SIRT1 shRNA Control, ***P < 0.001.

We next performed immunofluorescence analysis to confirm the Western blot results. Following the analysis, we found the SIRT1 Over-expression group to have increased formation of LC3 punctae, with the SIRT1 Control group having reduced LC3 dots (*n* = 3, *P* < 0.01; Figures 5A,B). Contrarily, the SIRT1 shRNA group had significant reduction of LC3 dots when compared to the SIRT1 shRNA Control group (*n* = 3, *P* < 0.001; Figures 5A,B).

## Discussion

Cerebral I/R injury is associated with a variety of cerebrovascular diseases, the most common type being stroke (19). OGD/R is a widely used *in-vitro* stroke model which shows similarities with the *in-vivo* model of brain ischemia (20). In this study, we established the *in-vitro* OGD/R model using PC12 cells to mimic cerebral I/R injury, and investigated the role of SIRT1 after OGD/R treatment. According to the time requirement of intravenous thrombolysis (21) and cell viability evaluated by the CCK8 assay (Figure 1), we selected 4 h for the OGD treatment to establish the OGD/R model. SIRT1 expression was regulated in PC12 cells by plasmids transfection, and the upper band in SIRT1 over-expression group corresponded to SIRT1-EGFP fusion protein (Figures 2A,B).

Apoptosis is paramount for cell survival during cerebral I/R (22). As an apoptotic-related gene, the *Bcl-2* gene family plays an important role in regulating apoptosis. Bcl-2 protein mainly binds to the outer membrane of the mitochondria and exerts an anti-apoptotic effect by inhibiting the release of cytochrome C to the cytosol and activating caspases. The contrary effect of this is exhibited by the pro-apoptotic protein, Bax (6). The ratio of these two proteins determines, in part, the susceptibility of cells to a death signal (23). In this study, we observed the inhibition of apoptosis when SIRT1 was up-regulated and the induction of apoptosis after SIRT1 down-regulation through the expression of Bcl-2 family members (Figures 2C–E). The anti-apoptotic effect of SIRT1 was further evidenced by TUNEL staining, which showed more cell death following SIRT1 down-regulation (Figures 3A,B). These results indicated that SIRT1 inhibited OGD/R-induced apoptotic cell death.

Autophagy is involved in cell survival, development, and death, where it acts as an adaptive response in providing nutrients and energy to cells. The role for SIRT1 in the induction of autophagy has been reported (24). Microtubule associated protein 1 light chain 3 (LC3), a homolog of yeast Atg8 in mammals, is a widely used autophagosome marker (25). LC3 is post-translationally cleaved and localized in the cytosol (LC3-I) or in the autophagosomal membranes (LC3-II) (15). Thus, the protein ratio, LC3-II/LC3-I, can be used to show the level of autophagy in the cell (26, 27). Beclin-1 is a mammalian ortholog of yeast Atg6 that is increased during programmed cell survival by inducing autophagy (28). P62/sequestosome-1 which is a multifunctional protein, has an LC3-interacting region (LIR) that is linked to autophagosome-localized protein LC3 during autophagy (29). This interaction is indispensable for the degradation of p62; thus, autophagy impairment is usually accompanied by significant accumulation of p62 (30). In our study, autophagy was promoted when up-regulating SIRT1 through the expression of Beclin-1 and the ratio of LC3-II/LC3-I (Figures 4D,E). The expression of LC3 which was quantified by LC3 punctae also supported our hypothesis that SIRT1 promotes autophagy in OGD/R-induced cell injury (Figure 5B). Moreover, P62 expression level was increased following SIRT1 down-regulation (Figure 4B), suggesting the impairment of autophagy. These results showed that SIRT1 promoted autophagy in OGD/R- induced PC12 cells.

Autophagy is important in cell death decisions and can protect cells by preventing them from undergoing apoptosis (31). Autophagy related gene 5 (*Atg5*) is involved in mammalian autophagosome formation and localization of other autophagy-related gene. Atg5 knock-out in mice led to organelle damage, disruption of energy balance, and early perinatal death (27). In this study, the expression of Atg5 was significantly increased when SIRT1 was down-regulated (Figure 4C), which could possibly be due to a cellular negative feedback. We also found the expressions of autophagy-related proteins to be lower in the Normal group than the other groups. This, we believe, might have been possibly due to the toxicity of the plasmid transfection. Furthermore, the autophagy-related proteins had a more significant change in the SIRT1 knock-down cells than the SIRT1 over-expressed cells, which means SIRT1 knock-down cells might have been more sensitive, suggesting the necessity of SIRT1 expression in OGD/R-induced PC12 cells.

Numerous studies consider SIRT1 as a protective factor in nerve system disease because it can modulate gene expression and adapt cell metabolism (32). Autophagy induced by SIRT1 prevents prion peptide neurotoxicity. Resveratrol, a SIRT1 agonist, has been shown to activate autophagy and reduce rotenone-mediated neurotoxicity (33, 34). SIRT1 promoted autophagy and reduced hypoxia-induced apoptosis to protect cardiomyocytes from hypoxic stress (35). On the basis of our experimental results, we hypothesize that an appropriate up-regulation of SIRT1 expression can promote autophagy and inhibit apoptosis in an OGD/R-induced injury, thereby increasing the tolerance of cells to OGD/R injury. The results from this study evidence the potential neuroprotective role of SIRT1 in cerebral I/R injury.

As this study was solely *in-vitro*, which is a potential limitation to this study, we will attempt to replicate this in an *in-vivo* experiment in the future. We will employ reversing verification method by using 3-methyladenine (3-MA) or chloroquine diphosphate salt (CQ) as an autophagy inhibitor, while also using mono-fluorescence GFP-LC3 and dual-fluorescence mRFP-eGFP-LC3 plasmids to transfect primary hippocampal neurons to measure the autophagic flux. Also, microRNAs will be used to mediate the cross-regulation between autophagy and apoptosis by targeting the key proteins involved in these two pathways.

## Data Availability Statement

The datasets generated for this study are available on request to the corresponding author.

## Author Contributions

MF and YG designed the experiments. QR and ZH drafted the manuscript. XT, YJ, GL, and NA performed the experiments and analyzed the data. BB revised the manuscript. All authors read and approved the final manuscript.

### Conflict of Interest

The authors declare that the research was conducted in the absence of any commercial or financial relationships that could be construed as a potential conflict of interest.

## Abbreviations

AKT: protein kinase B
AMPK: AMP-activated protein kinase
Atg: autophagy related gene
Bax: Bcl-2-associated X protein
BCA: bicinchoninic acid
Bcl-2: B-cell lymphoma 2
BSA: bovine serum albumin
CCK8: cell counting kit 8
FBS: fetal bovine serum
FOXO: forkhead box O
I/R: ischemia/reperfusion
IVT: intravenous thrombolysis
LC3: microtubule associated protein 1 light chain 3
LIR: LC3-interacting region
MAPK: mitogen-activated protein kinase
NAD: nicotinamide adenine dinucleotide
OGD/R: oxygen and glucose deprivation/reperfusion
P62: nucleoporin p62
PBS: phosphate buffer saline
PBST: phosphate buffer saline-Tween
PC12: pheochromocytoma 12
PI3K: phosphoinositide 3-kinases
RIPA: radio immunoprecipitation assay
RPMI: roswell park memorial institute
shRNA: short hairpin RNA
SIRT1: silent information regulator 1
TBST: Tris-HCl-Tween
TUNEL: terminal deoxynucleotidyl transferase mediated dUTP nick end labeling.
